# A Case of Sudden Unexpected Infant Death with Presumptive SARS-CoV-2 Infection

**DOI:** 10.3390/ijms27104604

**Published:** 2026-05-20

**Authors:** Veronika A. Galichina, Ruslan A. Nasyrov, Zlata V. Davydova, Simon E. Gabaraev, Orasmurad D. Yagmurov

**Affiliations:** 1 Department of Pathological Anatomy with a Course in Forensic Medicine Named After Professor D.D. Lokhov, Saint Petersburg State Pediatric Medical University, Ministry of Health of Russia, Saint Petersburg 194100, Russia; rrmd99@mail.ru (R.A.N.); zlata.davydova@rambler.ru (Z.V.D.); simongabaraev@mail.ru (S.E.G.); 2 Bureau of Forensic Medical Examination of the Leningrad Region, Saint Petersburg 195067, Russia; oraz.yagmurov@gmail.ru

**Keywords:** SUID, microvessels, IFN-γ, COVID-19

## Abstract

COVID-19 remains a challenge to the global healthcare despite the end of the pandemic, including due to the significant involvement of children in the epidemic process. During the pandemic period, an increase in the incidence of Sudden Unexpected Infant Death (SUID) and Sudden Infant Death Syndrome (SIDS) was observed. Currently, their rates remain elevated compared to the prepandemic period. The pathogenetic mechanisms underlying the fulminant course of infection in infants leading to fatal outcomes remain insufficiently understood. In this study, we report for the first time the results of histological and immunohistochemical examination of the lungs in a case of COVID-19-associated SUID in a 2-month-old infant. The absence of similar studies in the available literature limits opportunities for analyzing the pathogenesis of SUID. Our data allow a detailed characterization of the histological changes in the lungs, the localization and range of SARS-CoV-2 nucleocapsid protein expression, the identification of molecular mechanisms underlying apoptosis in the pulmonary microvascular endothelium, and the elucidation of the role of endothelial dysfunction. Particular attention in this article is devoted to the role of cytokines (IL-6, TNF-α, and IFN-γ) in the pathogenesis of hyperacute viral infection. The obtained data demonstrate substantial differences between the observed changes and the classic presentation of COVID-19 in older children. These findings offer prospects for improving prevention strategies and developing targeted therapy for fulminant forms of COVID-19, while also contributing to the understanding of SIDS pathogenesis.

## 1. Introduction

Novel coronavirus infection (COVID-19), caused by the SARS-CoV-2 virus, continues to represent a significant challenge to the global healthcare system, even several years after the conclusion of the pandemic. Initially, researchers focused their attention primarily on the adult population as the group at highest risk for severe disease and fatal outcomes. As data accumulated, it became evident that the virus affects all age groups, including children [1]. The consistent involvement of the pediatric population in the epidemic process, the presence of risks for severe course in young children and patients with comorbid conditions, as well as the clinical manifestations require the attention of practicing doctors.

Furthermore, some authors have reported an increased incidence of sudden unexpected infant death (SUID) during the pandemic. SUID (Sudden Unexpected Infant Death) and SIDS (Sudden Infant Death Syndrome) are terms associated with sudden deaths in infants under one year of age [2]. In cases of SUID, the immediate cause of death and a final diagnosis are established following autopsy. SIDS is diagnosed when a thorough death scene investigation, autopsy, and review of the patient’s history fail to reveal findings that would explain the fatal outcome [3]. A study by Guare et al. demonstrated that the risk of SUID and SIDS increased during the pandemic period (March 2020 to December 2021) compared to the prepandemic period (March 2018 to December 2019), with the most pronounced monthly increase observed from June to December 2021 [4]. D. Lisman et al. report that in children who died with a diagnosis of SIDS, SARS-CoV-2 antigens were detected postmortem by PCR [5]. Shapiro-Mendoza et al. have also reported an increase in SIDS cases during the COVID-19 pandemic, which they attribute to diagnostic shifting. The authors maintain that the available evidence does not support either a direct or an indirect impact of the 2019 coronavirus pandemic on the increased incidence of SUID [6]. Moreover, a case of SUID has been described in an infant with pronounced neurological symptoms and developmental delay, born to a mother who had contracted COVID-19 [7]. The PCR test for SARS-CoV-2 was negative in this infant at birth; however, SARS-CoV-2 nucleocapsid protein and spike glycoprotein 1 were detected in the mother’s placenta. Despite a sustained global decline in the number of SIDS cases over the past 30–40 years, the COVID-19 pandemic led to a statistical plateau in their incidence [8].

The contemporary morphological substrate of SIDS is represented by myocarditis, cardiomyopathies, and congenital heart defects [9]. According to the review by M. A. Sacco et al., the most frequent histopathological finding is lymphocytic myocarditis associated with DNA viruses (herpes group) or enteroviruses [9]. The use of immunohistochemical (IHC) staining with CD3 and CD45-R0 is effective for verifying the diagnosis of myocarditis, including in cases of SIDS [10]. Additional histological criteria for myocardial involvement include endocardial fibrosis of the atria and interventricular septum, expression of CD68 and CD117 in fibroblasts, mast cells, and lymphocytes, cardiomyocyte apoptosis (p53 expression), as well as fibroblast proliferation and cardiac remodeling (ki-67 expression) [11]. In cases of infectious etiology of SIDS, pronounced expression of CRP and ICAM-1 by hepatocytes, intense myocardial staining for CRP, and increased expression of IL-6 by hepatocytes (in cases caused by *E. coli*) are observed [12]. In our view, these observations should be classified as SUID, as the cause of death was ultimately established. Alongside cardiac causes, several authors have indicated the role of pathology in brainstem structures, including the retrotrapezoid nucleus, reticular formation, gigantocellular nucleus, substantia nigra, and hypoglossal nucleus of the medulla oblongata [13,14,15,16].

However, despite having seen some reports of a possible increase in the incidence of SUID in recent years, we found no data in the literature regarding morphological studies of cases with this diagnosis in the context of COVID-19. The emergence of new, severe forms of COVID-19 underscores the growing importance of investigating the pathogenesis of this condition. In this regard, a comparative pathogenetic assessment of pulmonary changes in cases of sudden infant death with COVID-19 is of particular relevance.

## 2. Case Presentation

Histological material was submitted to the Department of Pathological Anatomy with a Course in Forensic Medicine named after Professor D.D. Lokhov from the Bureau of Forensic Medical Examination for analysis and the provision of a consultative opinion regarding a case of sudden death in a 2.5-month-old infant. It is known that the infant was found at home with no external signs of violent death. A preliminary diagnosis of “Sudden Infant Death Syndrome?” and “Small-focal serous-desquamative pneumonia” had been made.

### 2.1. Patient

A 2 month old female infant was found deceased in the early morning of 15 December 2025, by her mother. At 04:30, the mother noted the absence of respiratory movements and the infant’s lack of response to touch. The mother called emergency medical services, who pronounced death. According to the medical history, the girl was born from her mother’s first pregnancy (the mother was 23 years old). The pregnancy was uncomplicated. Delivery was the first, at 40 weeks of gestation. The infant was born full term, mature, with spontaneous breathing, in satisfactory condition, and without any signs of infectious pathology or congenital malformations. Birth weight was 3060.0 g, length was 50.0 cm. Apgar scores were 8 and 9 at 1 and 5 min, respectively. The infant was discharged from the maternity hospital with her mother on day 6. The family appeared well during the home visit; the family was intact, and social conditions were normal. On the evening prior to death, the child’s condition caused no concern to the parents, and they reported no signs of respiratory or other infections. The infant was sleeping in a separate crib in an adjacent room. The girl was found lying supine, lightly clothed. Pillows were placed away from the child. The parents had no symptoms of acute respiratory viral infection. No outside individuals had been in contact with the child.

At autopsy, the coroner observed reddish frothy fluid in the nasal passages and oral cavity. Macroscopically, on cut section, the lung tissue appeared variegated due to alternating dark red and gray-red areas; the tissue consistency was doughy, with a firm texture predominantly at the lung hila and in the lower lobes. The cut surface was moist, with a large amount of pink-red frothy fluid exuding from the incised tissue, and a moderate amount of dark red liquid blood exuded from the incised vessels.

Postmortem PCR testing of the trachea and lung was performed. Infections caused by the following pathogens were ruled out (negative PCR results): SARS-CoV-2, *Mycoplasma pneumoniae*, *Chlamydophila pneumoniae*, Respiratory Syncytial virus, Parainfluenza virus types 1, 2, 3, and 4, Human Coronavirus (hCoV), Rhinovirus, Adenovirus groups B, C, and E, Bocavirus, and Influenza A/B virus.

### 2.2. Materials and Methods

We analyzed the autopsy report and the postmortem specimens of the infant. Ethical clearance was obtained from the Local Ethics Committee of the St. Petersburg State Pediatric Medical University, under protocol number 66/10, approved on 20 March 2026.

Paraffin sections were stained using hematoxylin and eosin and Masson’s trichrome.

An immunohistochemical investigation was performed. The following primary antibodies were used: polyclonal antibodies of nucleocapsid SARS-CoV-2 (manufactured by GeneTex, Irvine, CA, USA); monoclonal antibodies of CD20 (L26 clone, manufactured by Diagnostic BioSystems, Pleasanton, CA, USA); monoclonal antibodies of CD8 (144B clone, manufactured by Diagnostic BioSystems, Pleasanton, CA, USA); monoclonal antibodies of CD31 (Jc/70A clone, manufactured by Diagnostic BioSystems, Pleasanton, CA, USA); polyclonal antibodies of CD95 (manufactured by Diagnostic BioSystems, Pleasanton, CA, USA); monoclonal antibodies of CD4 (4B12 clone, manufactured by Dako, Glostrup, Denmark); monoclonal antibodies of CD68 (KP1 clone, manufactured by Diagnostic BioSystems, Pleasanton, CA, USA); polyclonal antibodies of IL-6 (manufactured by Bio-Rad, Hercules, CA, USA); monoclonal antibodies of TNF-α (DBM15.28 clone, Diagnostic BioSystems, Pleasanton, CA, USA); monoclonal antibodies of IFNγ (IFNG/466 clone, GeneTex, Irvine, CA, USA); polyclonal antibodies of fibrinogen (manufactured by DakoCytomation, Glostrup, Denmark). The Mouse/Rabbit UnoVue HRP/DAB Detection System (manufactured by Diagnostic BioSystems, Pleasanton, CA, USA) was used for the IHC procedure.

## 3. Results

**Figure 1:**
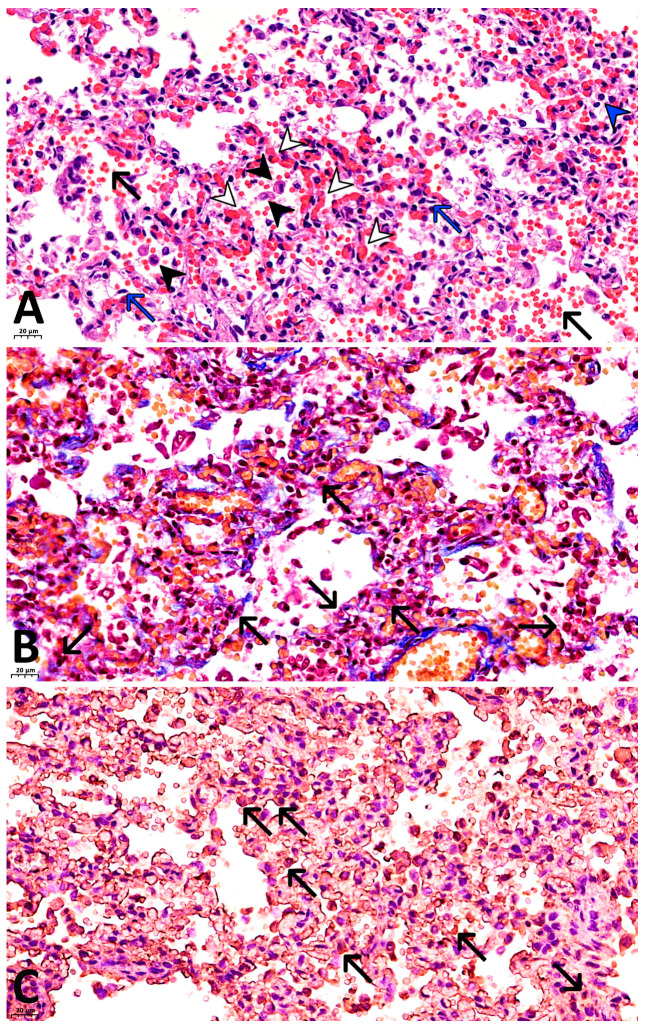
Microvessels of the lungs in COVID-19. (**A**) Endothelial desquamation (blue arrows), intra-alveolar hemorrhages (black arrows), multiple macrophages in the alveolar lumens (black arrowheads), focal lymphocytic infiltration (blue arrowhead), congestion and stasis of erythrocytes and thrombosis of the interalveolar septal capillaries (white arrowheads) were observed in a patient with COVID-19. H&E staining, ×400; (**B**) Thrombosis of the interalveolar septal capillaries with fibrin thrombi (black arrows), fibrin strands in the alveolar lumens. Staining by trichrom according to Masson’s method, ×400; (**C**) Fibrin clots in the capillaries of the interalveolar septa (black arrows), fibrin deposits in the form of strands along the inner surface of the alveoli and along the apical part of the bronchial epithelial cells. IHC with fibrinogen, ×400.

Microscopic examination revealed signs of microcirculatory vessel damage, characterized by focal endothelial desquamation. The remaining endothelium consisted of elongated cells with hyperchromatic oval nuclei arranged parallel to the basement membrane. Alongside this, swollen endothelial cells oriented perpendicular to the vascular wall were observed, as well as mild perivascular edema. Arterioles and venules were congested, with the presence of erythrocyte thrombi (Figure 1A). Capillaries of the interalveolar septa were markedly congested, with erythrocyte stasis and fibrin, erythrocyte, and mixed thrombi identified in the majority of them (Figure 1B,C). Hemorrhagic foci were visualized within the interalveolar septa.

**Figure 2:**
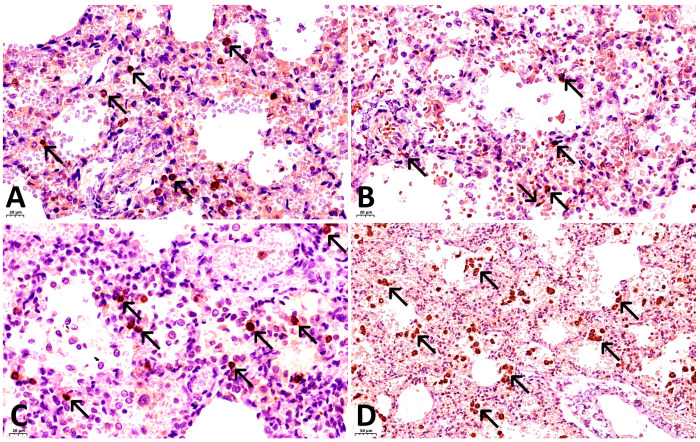
Pulmonary inflammatory infiltration. (**A**) Infiltration by CD8+ positive T-lymphocytes (black arrows). IHC with CD8, ×400; (**B**) Mild infiltration by CD4+ positive T-cells (black arrows). IHC with CD4, ×400; (**C**) Infiltration by numerous CD20+ positive B-lymphocytes (black arrows). IHC with CD20, ×400; (**D**) Abundant macrophages in the alveolar lumina and interalveolar septa (black arrows). IHC with CD68, ×200.

Foci of inflammatory lymphocytic infiltration, composed of T- and B-lymphocytes, were observed within the interalveolar septa in all examined areas of lung tissue (Figure 2A–C). Isolated small areas of atelectasis were identified. Focal desquamation of alveolocytes was noted. Some alveoli were filled with fresh blood. Fibrin strands were visualized in the lumina of several alveoli, with initial signs of fibrin aggregation observed in scattered alveoli. Additionally, large macrophages were present in some alveoli (Figure 2D). Bronchial epithelium exhibited focal desquamation.

Masson’s trichrome staining confirmed thrombosis of the pulmonary capillary network, with mixed and fibrin thrombi (Figure 1B).

**Figure 3:**
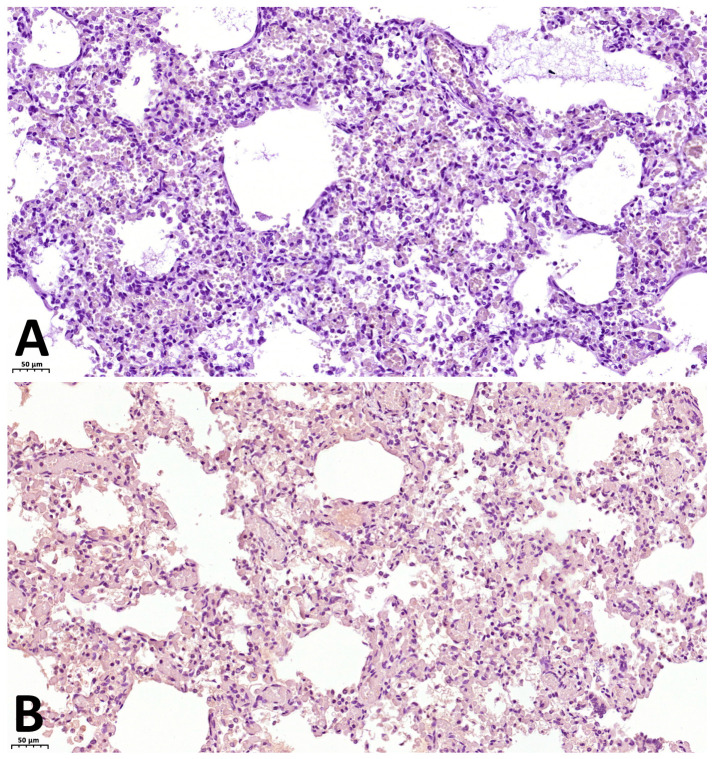
A negative control for SARS-CoV-2 nucleocapsid staining: (**A**) with phosphate buffer (pH 7.6) instead of antibodies against SARS-CoV-2 nucleocapsid, ×200; (**B**) IHC with SARS-CoV-2 nucleocapsid in patient with Viral pneumonia of non-COVID-19 etiology, ×200.

**Figure 4:**
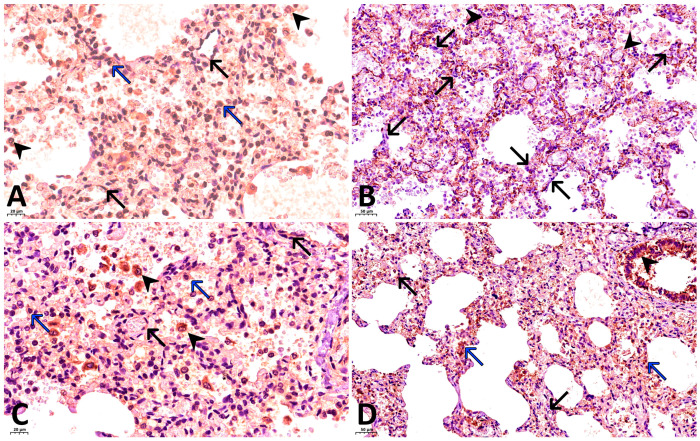
Other changes in patient’s lungs. (**A**) Pronounced expression of SARS-CoV-2 nucleocapsid molecules in the endothelium of pulmonary microvessels (black arrows), in macrophages (black arrowheads) and alveolar cells (blue arrows). IHC, ×400; (**B**) CD31 expression is observed in the endothelium of the microvasculature and the capillaries of the interalveolar septa. Discontinuity of the pulmonary capillary network (black arrows), focal loss of marker expression (black arrowheads) are noted. IHC with CD31, ×200; (**C**) Strong IFN-γ expression in alveolar macrophages (black arrowheads), alveolar epithelial cells (blue arrows), and microvascular endothelium (black arrows). IHC with IFN-γ, ×400; (**D**) Significant CD95 expression was detected in the endothelium of the microvasculature (black arrows), the capillaries of the interalveolar septa (blue arrows), and in alveolar and bronchial epithelial cells (black arrowhead). IHC with CD95, ×200.

Results of immunohistochemical (IHC) staining:

- With antibodies against fibrinogen: focal fibrin deposits are observed as strands along the inner surface of the alveoli, along the apical portion of bronchial epithelial cells, as well as fibrin thrombi in the capillaries of the interalveolar septa (Figure 1C).
- With antibodies against CD68: marked expression of the marker is observed, with 30–40 macrophages visualized per high-power field (×400), and their number increasing to 50 or more in clusters (Figure 2D).
- With antibodies against CD8: 10–15 T lymphocytes are identified per high power field (×400), with their concentration increasing to 30–35 cells within lymphocytic aggregates (Figure 2A).
- With antibodies against CD20: 15–20 B lymphocytes are identified per high power field (×400), increasing to 30–35 or more cells within lymphocytic aggregates (Figure 2C).
- With antibodies against CD4: 3–6 T lymphocytes are identified per high power field (×400), with focal increase to 8–10 cells (Figure 2B).
- As a first negative control for SARS-CoV-2 nucleocapsid staining, phosphate buffer (pH 7.6) was applied to the paraffin section of the lung from the investigated case instead of antibodies against this antigen. No expression was observed on these sections (Figure 3A). For the second negative control, lung tissue from a child with viral pneumonia who died from causes other than COVID-19 was used. In this specimen, expression was likewise absent (Figure 3B).
- With antibodies against COVID 19 nucleocapsid protein: markedly pronounced expression (+++) was detected in the endothelium of microcirculatory vessels, bronchial epithelium, alveolar macrophages, and alveolocytes (Figure 4A).
- With antibodies against IL-6: no expression was detected (negative, confirmed twice).
- With antibodies against TNF-α: no expression was detected (negative, confirmed twice).
- With antibodies against CD31: the capillary network demonstrates a discontinuous pattern, with focal loss of marker expression in the endothelium of some capillaries, and areas of absent expression fields are noted. At the same time, marked expression (+++) is focally observed in the endothelium of microcirculatory vessels (Figure 4B).
- With antibodies against IFN-γ: marked expression (+++) is observed in the endothelium of microcirculatory vessels, bronchial epithelium, alveolocytes, and alveolar macrophages (25–30 cells per high power field at ×400 magnification) (Figure 4C).
- With antibodies against CD95: moderately pronounced expression (++) is observed in the endothelium of some microcirculatory vessels, as well as marked expression (+++) in the bronchial epithelium, alveolar macrophages, and alveolocytes (Figure 4D).

## 4. Discussion

The present study reports a case of sudden unexpected infant death presumably with COVID-19. Histological changes in the lung tissue were investigated using IHC analysis. During the study, morphological features characteristic of the early period of the exudative phase of diffuse alveolar damage in COVID-19 were identified. In the present case, band-like hyaline membranes did not form on the alveolar walls, in contrast to our previously described observation (a child aged 1 year and 5 months, with a disease duration of 5 days) [17]. Fibrin deposits were observed as strands along the inner surface of the alveoli and along the border of bronchial epithelial cells. At the same time, more intense inflammatory infiltration with a predominance of T- and B-lymphocytes was documented. Markedly pronounced expression of the SARS-CoV-2 nucleocapsid protein, detected in the microvascular endothelium, bronchial epithelium, alveolocytes, and macrophages, was a notable feature, which also distinguishes this observation from the previously mentioned case. The immunohistochemical reaction product was detected diffusely across the entire surface of the section.

PCR tests are characterized by high sensitivity; however, cases of false-negative results in the diagnosis of COVID-19 are still being reported. Available evidence suggests that the prevalence of such results is heterogeneous and varies depending on study design [18,19]. According to various authors, the proportion of false-negative results may reach 33–42% [20,21]. Various causes of false-negative results in SARS-CoV-2 testing are described in the literature. Thus, F. Sessa and colleagues highlight the presence of a “window” period at the initial phase of infection, wherein the quantity of viral RNA remains beneath the detection capability of the diagnostic test [22]. D. Keaney et al. report that PCR tests performed on bronchoalveolar lavage material have greater diagnostic accuracy [23]. D. Lisman and co-authors observed an inverse correlation between viral expression in the nasopharynx and the lungs: lower nasopharyngeal viral expression is associated with higher expression in pulmonary tissue [5]. Furthermore, viral mutations may affect RNA detection, particularly in cases where the test system primers do not match new variants [24]. Additionally, test sensitivity is dependent upon several preanalytical, human, and physical factors beyond molecular technology itself: insufficient specimen volume [18], compromised temperature storage conditions, delays in storage and processing [25,26], and the timing of specimen collection in relation to the phase of infection [27,28]. High-quality specimen collection requires specific training and technical knowledge. The high frequency of false-negative results casts doubt on the absolute reliability of PCR testing. It is worth noting that the IHC method is also not without certain limitations (e.g., background staining, sectioning and fixation artifacts, and the inability to distinguish active infection from residual persisting antigen). Nevertheless, IHC has demonstrated high significance, reliability, and promise, for instance, in oncomorphology and oncology [29]. Despite the greater number of factors that compromise analytical quality in postmortem examinations, IHC detection of SARS-CoV-2 nucleocapsid molecules was consistent with positive PCR results from both antemortem and postmortem testing in children as well as adults. In one of the cases described by us, SARS-CoV-2 was detected by antemortem PCR testing, whereas postmortem PCR examination of bronchial and lung tissue material did not reveal the virus. The viral etiology of the infection was confirmed only through the use of IHC staining [17]. Nevertheless, the discussion regarding verification of the causative agent remains open.

A negative result from postmortem PCR testing does not completely rule out COVID-19 in the child within this study. The results of PCR testing should be interpreted with caution, taking into account laboratory and instrumental findings. The very fact of SUID according to the epidemiological pattern mentioned earlier, the nature of morphological changes in the lungs, the pattern of CD31 expression (discontinuity of the capillary network), and CD95 expression (pronounced apoptosis) allow for the suspicion of novel coronavirus infection. The detection of marked expression of SARS-CoV-2 nucleocapsid molecules by IHC increases the likelihood of COVID-19 in the child.

The obtained results confirm previously established patterns of COVID-19 pathogenesis: (1) involvement of endothelial and epithelial cells as the main target cells; (2) pronounced expression of viral antigen in macrophages. The high density of macrophages per high-power field was noteworthy. This concentration of macrophages significantly exceeded the density of macrophages in the lungs of the child with a 5-day disease duration. The cells were distributed both diffusely and as focal aggregates in the interstitium and alveolar lumina. Staining using CD31 (an endothelial marker) revealed destructive changes in the vessel wall. This finding is further supported by the colocalization of the apoptosis marker CD95 and the SARS-CoV-2 antigen in the endothelium. This allows us to conclude that the defining mechanism of interaction in the “virus host” system in COVID-19 is the induction of apoptosis. Apoptosis of endothelial cells represents the primary pathway of their death. This is consistent with the results of previous studies [17,30].

Analysis of the cytokine profile is of substantial importance for understanding the pathogenesis and morphogenesis of sudden infant death. It is known that several cytokines, including IL-6, TNF-α, and IFN-γ, correlate with the severity of COVID-19 [31,32,33,34,35], serving as independent predictors of unfavorable outcomes. The role of IFN-γ is of particular interest in the context of the obtained data. As a key cytokine of antiviral immunity [36], IFN-γ, on one hand, suppresses viral replication, and on the other, enhances the activity of cytotoxic T-lymphocytes [37]. According to the literature, high concentrations of IFN-γ are associated with severe COVID-19 [38]. Statistically significant higher levels of IFN-γ were documented in cohort studies among patients with fatal outcomes [39]. Hyperproduction of IFN-γ in serum correlates with a favorable prognosis in only isolated reports. Among the pleiotropic effects of IFN-γ is the enhancement of cellular sensitivity to apoptosis through stimulation of the Fas/FasL system expression [40]. This likely explains the markedly positive expression of the apoptosis marker CD95 and the development of destructive changes in the microvascular endothelium in the presented case. The complexity of interpreting the pathogenesis in this case is due to the fact that, on one hand, IFN-γ suppresses viral replication and stimulates cytokine production by T-lymphocytes [37]; on the other hand, in the presented case of the deceased 2-month-old infant, markedly pronounced expression of SARS-CoV-2 antigens was observed in the absence of TNF-α and IL-6 expression. It should also be added that in our previous studies of children who died from COVID-19, moderately pronounced expression of IL 6 molecules was detected in microvessels, pulmonary macrophages, and bronchial epithelium [30].

The age factor appears to be of considerable importance in this context. It has been established that the expression level of genes related to the canonical IFN-γ pathway in patients with COVID-19 decreases with age [41], whereas the quantity of neutralizing autoantibodies against interferon-I increases with age and also leads to a reduction in IFN-γ levels [42]. Studies indicate that IFN-γ levels in children with COVID-19 are elevated, but do not reach the values characteristic of adults. According to the authors, this may suggest a less severe course of infection in the pediatric population [43]. Alterations in IFN-γ expression are observed during the cytokine storm induced by SARS-CoV-2 [39]. It has been suggested that the increased severity of infection in adults may be related to hyperproduction of cytokines and reduced immunomodulatory capacity, whereas limited IFN-γ production by T-cells in infants and increased numbers of CD8+ T-cells in older children may confer a protective effect [44]. A sustained humoral response develops in infants, and limited IFN-γ production in response to SARS-CoV-2 likely prevents the development of severe forms of COVID-19 [45]. The potential role of IFN-γ, particularly in the early stages of SARS-CoV-2 infection, may partly determine the variability of clinical manifestations—from asymptomatic course to severe forms [46]. Hyperproduction of IFN-γ molecules can induce an excessive immune response, known as a cytokine storm [47]. Summarizing the literature data, it can be concluded that the markedly increased expression of IFN-γ in the lung tissue of the infant with presumptive COVID-19 likely induced apoptosis of endothelial cells, resulting in the formation of multiple thrombi within the lumina of pulmonary capillaries.

The presumed course of COVID-19 in this patient can be characterized as hyperacute (fulminant) based on the medical history data and the nature of histological and immunohistochemical findings.

## 5. Conclusions

In this case of sudden unexpected infant death, the key pathogenetic links determining the mechanism of COVID-19 progression can be traced: infection of the pulmonary microvascular endothelium—hyperexpression of IFN-γ molecules in the microvascular endothelium and macrophages—induction of apoptosis—endothelial cell death—thrombosis of pulmonary capillaries. The alveolar-capillary block and disseminated intravascular coagulation (DIC) syndrome, as the immediate cause of death, develop subsequently.

The analysis of the results was conducted within the framework of our proposed microvascular theory of pathology [30]. This allowed for a pathogenetic assessment of virus-host interaction in this course of infection. The obtained data expand the understanding of the pathogenesis of viral infections and may prove useful in elucidating pathologies that remain unknown, such as sudden infant death syndrome. Elucidating the causes of dysregulation of cytokine expression, as well as their prevention and correction in COVID-19, is of critical importance.

## Data Availability

The original contributions presented in the study are included in the article, further inquiries can be directed to the corresponding author.

## Abbreviations

The following abbreviations are used in this manuscript:

SUID	Sudden Unexpected Infant Death
SIDS	Sudden Infant Death Syndrome
IL-6	Interleukin-6
TNF-α	Tumor Necrosis Factor-α
IFN-γ	Interferon-γ
PCR	Polymerase Chain Reaction
DNA	Deoxyribonucleic Acid
IHC	Immunohistochemical
CD	Cluster of Differentiation
CRP	C-Reactive Protein
ICAM-1	Intercellular Adhesion Molecule 1
H&E	Hematoxylin and eosin
DIC	Disseminated intravascular coagulation
